# Computational generation of proteins with predetermined three-dimensional shapes using ProteinSolver

**DOI:** 10.1016/j.xpro.2021.100505

**Published:** 2021-04-28

**Authors:** Alexey Strokach, David Becerra, Carles Corbi-Verge, Albert Perez-Riba, Philip M. Kim

**Affiliations:** 1 Department of Computer Science, University of Toronto, Toronto, ON M5S 3E1, Canada; 2 Donnelly Centre for Cellular and Biomolecular Research, University of Toronto, Toronto, ON M5S 3E1, Canada; 3 Department of Molecular Genetics, University of Toronto, Toronto, ON M5S 3E1, Canada

**Keywords:** Bioinformatics, Biophysics, Circular Dichroism (CD), Protein Biochemistry, Protein expression and purification, Structural Biology

## Abstract

Computational generation of new proteins with a predetermined three-dimensional shape and computational optimization of existing proteins while maintaining their shape are challenging problems in structural biology. Here, we present a protocol that uses ProteinSolver, a pre-trained graph convolutional neural network, to quickly generate thousands of sequences matching a specific protein topology. We describe computational approaches that can be used to evaluate the generated sequences, and we show how select sequences can be validated experimentally.

For complete details on the use and execution of this protocol, please refer to Strokach et al. (2020).

## Highlights

• ProteinSolver can generate amino acid sequences of proteins with specific shapes
• We describe how to validate and rank generated sequences using computational approaches
• We describe how to express, purify, and validate generated proteins using CD

## Graphical abstract

**Figure:**
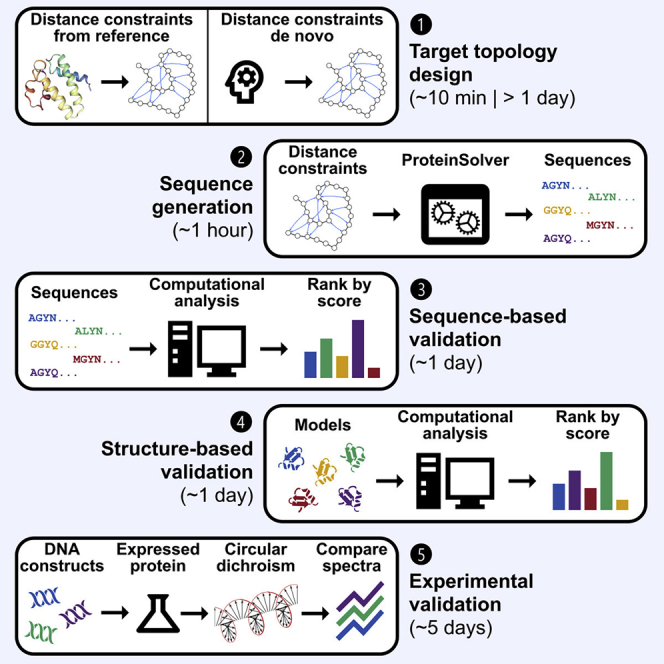

## Before you begin

### Confirm that ProteinSolver is the best tool for your task

**Timing: 5 min**

ProteinSolver is a graph neural network capable of generating novel amino acid sequences that fold into proteins with predetermined topologies. ProteinSolver was trained using millions of amino acid sequences and corresponding distance matrices, and its predictions have been validated both computationally and experimentally (Strokach et al., 2020).

Some applications where ProteinSolver could be especially useful include:

1. Generating libraries of amino acid sequences that fold into proteins with specific topologies. Such libraries would have a much higher probability of containing stable proteins with desired properties than libraries generated using traditional approaches such as random mutagenesis.
2. Designing proteins that meet specific objectives, such as having low sequence identity but the same fold or having high sequence identity but different folds.
3. Finding positions and residues that are most important for maintaining a given protein fold.

However, ProteinSolver may not be the best tool for all problems in protein design. In particular:

4. ProteinSolver is not designed for protein structure prediction. If your goal is to predict the structure corresponding to a given protein sequence or distance matrix, tools such as Modeller (Webb and Sali, 2002) and trRosetta (Yang et al., 2020) may be more appropriate.
5. ProteinSolver is not designed for finding *existing* protein sequences with high sequence similarity to a query protein. If your goal is sequence similarity search, tools as BLAST+ (Camacho et al., 2009), hhsearch (Steinegger et al., 2019), and mmseqs2 (Steinegger and Söding, 2017) may be more appropriate.
6. ProteinSolver is not designed for finding *existing* protein structures with high structural similarity to a query protein or topology. If your goal is structure similarity search, tools such as CLICK (Nguyen et al., 2011) or VAST+ (Madej et al., 2014) may be more appropriate.

### Obtain a distance matrix or a protein structure with the target protein geometry

**Timing: 10 min**

7. In order to use ProteinSolver, you need a distance matrix, or a protein structure, corresponding to the target protein geometry. If there already exists a structure of a protein with the target geometry in the PDB, it is best to simply provide that structure as input. If the target geometry is novel, you need to create a file listing the shortest distances between all pairs of residues within 12 Angstrom of each other (see materials and equipment).

**CRITICAL:** ProteinSolver was trained using distance matrices extracted from structural templates in the PDB. While ProteinSolver should be robust to minor errors and gaps in the provided distance matrix, using a distance matrix that does not correspond to an actual protein topology, or is missing many edges, may lead to undefined behavior.

### Install software

**Timing: 2 h**

ProteinSolver is accessible through a web server, and no additional dependencies need to be installed in order to generate sequences for a particular protein topology. However, it may be advisable to analyze the generated sequences using several external tools and rank them by their stability or physicochemical properties. In steps 8–14 of this protocol, we detail the use of external tools to validate the generated sequences. Those tools can be installed on Linux and Windows Subsystem for Linux 2 (WSL2), and most can also be installed on MacOS.

8. We recommend using the conda package manager to install all dependencies that are available as conda packages.
  a. Download and install miniconda by following instructions on the miniconda downloads page: https://docs.conda.io/en/latest/miniconda.html.
  b. In order to be able to create homology models, you will need to obtain a MODELLER license key by following instructions on the MODELLER website: https://salilab.org/modeller/registration.html. Set the KEY_MODELLER environment variable to the license key that you obtain before installing the modeller conda package.
  c. Use the following system command to create a “proteinsolver” conda environment containing most of the software packages that are used in this protocol.

**System command that installs all packages available through conda**

export KEY_MODELLER=XXXXX
conda create –n proteinsolver –c ostrokach-forge –c salilab –c conda-forge –c defaults \
  proteinsolver \
  biopython \
  psipred \
  mdtraj \
  modeller

9. If you wish to use Rosetta to evaluate the stability of generated proteins, you need to download the precompiled Rosetta binaries from the Rosetta Commons website: https://www.rosettacommons.org/software/academic/. A Rosetta license can be obtained free of charge for academic use.
10. In order to perform molecular dynamics simulations, you will need to obtain a license key and download Amber by following instructions on the Amber website: https://ambermd.org/.

## Key resources table

**Table:** 

REAGENT or RESOURCE	SOURCE	IDENTIFIER
**Bacterial and virus strains**		

*E. coli* OverExpress C41(DE3) (Green cap)	Lucigen	Cat#60442-2

**Critical commercial assays**		

Pierce BCA Protein Assay Kit	Thermo Fisher	Cat#23227

**Deposited data**		

Protein Data Bank (PDB)	(Berman et al., 2000)	ftp://ftp.wwpdb.org/pub/pdb/data/structures/divided/
UniParc	(UniProt Consortium, 2019)	http://www.uniprot.org/downloads

**Recombinant DNA**		

Plasmid: pRSET-A	Thermo Fisher	Cat#V35120

**Software and algorithms**		

PyTorch	(Paszke et al., 2017)	https://github.com/pytorch/pytorch
PyTorch Geometric	(Fey and Lenssen, 2019)	https://github.com/rusty1s/pytorch_geometric
MDTraj	(McGibbon et al., 2015)	https://github.com/mdtraj/mdtraj
SciPy	(Virtanen et al., 2020)	https://github.com/scipy/scipy
Biotite	(Kunzmann and Hamacher, 2018)	https://github.com/biotite-dev/biotite
Amber 16	(Case et al., 2005)	https://ambermd.org/
PSIPRED	(Jones, 1999)	http://bioinf.cs.ucl.ac.uk/psipred/
MODELLER	(Webb and Sali, 2002)	https://salilab.org/modeller/
Rosetta	(Alford et al., 2017)	https://www.rosettacommons.org/software/
I-TASSER	(Yang et al., 2015)	https://zhanglab.ccmb.med.umich.edu/I-TASSER/
Quark	(Xu and Zhang, 2012)	https://zhanglab.ccmb.med.umich.edu/QUARK/
BeStSel	(Micsonai et al., 2015)	http://bestsel.elte.hu/index.php

## Materials and equipment

***Alternatives:*** In this protocol, we provide as input to ProteinSolver a PDB file containing the structure of a protein with the target protein topology. Alternatively, we could provide a TXT file listing the distances between all pairs of residues that are within 12 Ångström of each other, as show in the table below. In this TXT file, the first line in the file should start with “N: ” followed by the number of residues in the target protein, while all subsequent lines should provide the index of the first interacting residue (starting from 0), the index of the second interacting residue (starting from 0), and the shortest distance between the residues (in Ångström). Lines that start with a “#” are treated as comments and are ignored.

**Text file describing the target protein topology**

# Number of residues in sequence
N: 92
.
# Index of residue 1, Index of residue 2, Distance between residues (in A)
0, 1, 1.3283
0, 2, 3.5510
0, 3, 7.1788
0, 4, 9.1295
0, 5, 7.0669
1, 2, 1.3257
1, 3, 4.5414
1, 4, 5.3692
1, 5, 3.6796
…

***Alternatives:*** In this protocol, we refer to the ProteinSolver web server available at https://design.proteinsolver.org. If you prefer to self-host the web server, for example due to privacy or compliance reasons, we also provide a Docker image containing ProteinSolver and all components required to run the web server (see https://gitlab.com/ostrokach/proteinsolver/container_registry). First, you need to download and install Docker (see the official installation guide here: https://docs.docker.com/get-docker/). Next, you need to download and start the ProteinSolver Docker image. On Linux and Windows Subsystem for Linux 2 (WSL2), this can be accomplished running the following system commands. Once the ProteinSolver Docker image is running, the web server would be accessible at: http://localhost:8080.

**System command required to start the ProteinSolver web server locally**

$ export PORT=8080
$ export NOTEBOOK_PATH=proteinsolver/notebooks/30_design_dashboard.ipynb
$ docker run \
  --publish ${PORT}:${PORT} \
  --env=PORT=${PORT} --env=NOTEBOOK_PATH=${NOTEBOOK_PATH} \
  --gpus all \
registry.gitlab.com/ostrokach/proteinsolver:v0.1.24

***Alternatives:*** We use Invitrogen’s GeneArt platform to perform codon optimization and order synthetic gene constructs (see step 15). However, many other companies provide gene synthesis services, including GeneScript, GENEWIZ, IDT, Synbio Technologies, and Twist bioscience.

***Alternatives:*** We use the BugBuster Master Mix (Millipore) in order to extract protein expressed by E. coli cells (see step 16). However, one could use another commercial lysis buffer, such as the B-PER Complete Bacterial Protein Extraction Reagent (ThermoFisher), or the more traditional mechanical lysis methods, such as sonication and the French pressure cell press.

***Alternatives:*** We use dialysis to resuspend the eluted protein in a PBS buffer (see step 17). However, desalting columns are a viable alternative and could be used to purify and resuspend the eluted protein quicker and using less buffer, although with a slightly lower yield.

## Step-by-step method details

### Generation of amino acid sequences using ProteinSolver

**Timing: 1 h**

ProteinSolver (Strokach et al., 2020) is a graph convolutional neural network capable of generating amino acid sequences that fold into proteins with pre-determined geometric shapes. ProteinSolver takes as input a reference structure or a sparse distance matrix, listing distances between pairs of residues that are within 12 Å of one another, and a protein sequence, where one or more residues to be optimized are masked. The outputs of ProteinSolver are filled sequences, where masked residues are replaced with amino acids deemed to be best suited for their respective positions in the protein. ProteinSolver can be used for computational generation of new proteins with a predetermined three-dimensional shape as well as for computational optimization of existing proteins that maintains their shape.

**Figure 1:**
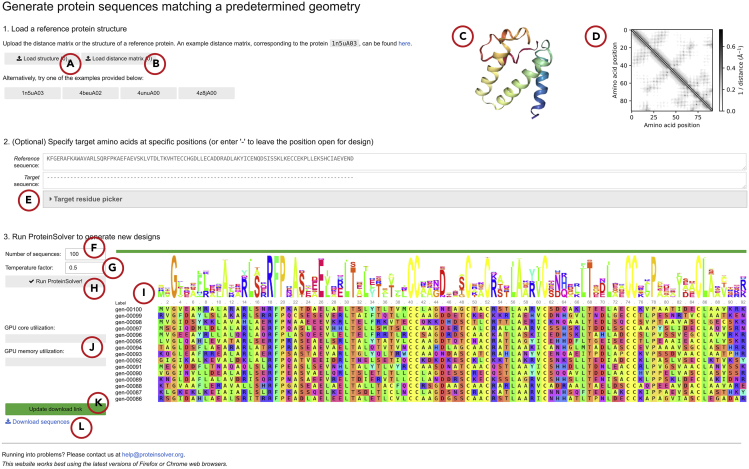
Screenshot of the ProteinSolver design web server (http://design.proteinsolver.org) (A) The user can upload the structure of a reference protein whose geometry will be used to restrain the space of generated amino acid sequences. The user can also select one of four example proteins. (B) Alternatively, the user can upload a sparse distance matrix describing the topology of the target protein (see materials and equipment). (C) The structure preview panel shows the structure of the reference protein uploaded by the user. (D) The topology preview panel shows the distance matrix of the target topology specified by the user. (E) The user can explicitly fix one or more amino acids in the generated sequences to specific residues. (F) The user can control the number of sequences generated in each run by changing the value in the “Number of sequences:” field. (G) The user can control the temperature factor used to generate sequences by changing the value in the “Temperature factor:” field. (H–L) (H) When the user clicks the “Run ProteinSolver” button, a background ProteinSolver process starts generating sequences matching the specified geometric and amino acid constraints. The progress of the ProteinSolver process can be monitored by looking at the progress bar and the sequence logo displayed to the right of the “Run ProteinSolver” button (I), while GPU utilization can be monitored by looking at the status bars displayed below the “Run ProteinSolver” button (J). Once a sufficient number of sequences have been generated, the user can click the “Generate download link” button (K), at which point a download link will appear (L), allowing the user to download the generated designs.
3. If you want to pre-assign certain amino acids to certain positions in the target protein, click the “Target residue picker” button (Figure 1E). Then, use the drop-down menus for each position that you want to constrain to select the desired amino acid. Your changes should be reflected in the “Target sequence:” panel above the “Target residue picker”. This feature is especially useful in cases where you know *a priori* that certain residues are essential for a specific protein function, such as catalytic activity and binding to other molecules, that you wish to preserve.
4. Select the number of sequences that you would like ProteinSolver to generate (Figure 1F). We recommend generating between 100 and 5000 sequences per round. If more sequences are required, you can run ProteinSolver multiple times, as different random seeds are used for each run.
5. Select the “temperature factor” that you would like to use when generating sequences (Figure 1G). The temperature factor controls how likely ProteinSolver is to sample low-probability sequences relative to high probability sequences. With a temperature factor of 1, ProteinSolver will use probabilities that achieved the lowest loss during training. With a higher temperature factor, ProteinSolver will be relatively more likely to select low-probability amino acids, while with lower temperature factor, ProteinSolver will be relatively less likely to select lower probability amino acids. We recommend using a temperature factor of 0.01 when you aim to generate a small number of high confidence sequences, and a temperature factor of 0.1 to 1 when you aim to generate a large number of sequences that will be filtered and refined in subsequent steps.
6. Click “Run ProteinSolver!” (Figure 1H) to start generating sequences. The panel on the right (Figure 1I) should show a live preview of the sequences that are generated and their corresponding sequence logo. The GPU monitoring panel (Figure 1J) should show increased GPU usage.
7. Once ProteinSolver stops running, click “Generate download link” button (Figure 1K) to generate a “Download sequences” link (Figure 1L) that will allow you to download a FASTA file listing generated sequences.

***Note:*** The header of every entry in the FASTA file has the following format: >{sequence_index}|{unique_name}|{sequence_identity}|{avg_logprobability}. The sequence_index field is a monotonically increasing index of the sequence in the FASTA file. The unique_name field is a unique name assigned to each sequence in the FASTA file. The sequence_identity field lists the sequence identity between the generated sequence and the sequence of the reference protein, if provided. The avg_logprobability field lists the average log-probability assigned by ProteinSolver to amino acids in the generated sequence.

1. Navigate to the ProteinSolver web server, accessible at https://design.proteinsolver.org. In some cases, the web server may take up to several minutes to load, as system resources are provisioned in proportion to demand.
2. Click the “Load structure” button to load the structure of a protein with the reference topology (Figure 1A). Alternatively, click the “Load distance matrix” button to load the distance matrix describing the reference topology (Figure 1B).
  a. A valid distance matrix, corresponding to CATH domain: 1n5uA03, is provided as an example above the “Load distance matrix” button.
  b. If you provide the structure of a reference protein by clicking the “Load structure” button, that structure should appear in the structure preview panel (Figure 1C).
  c. The distance matrix corresponding to the target topology should appear in the distance matrix preview panel (Figure 1D).

**Example FASTA file produced by the ProteinSolver web server**

>0|gen-00001|0.28875115513801575|-1.845043659210205
FFGYDFLREDAHAFLANRFPYASQAHIAKITACETECCNCCNERDLKSCTDCKNTLAQEICKNRERFSEEIGNCCENPPH
IIAECLAQVKHR
>1|gen-00002|0.29804882407188416|-1.976570963859558
FAGLKDDLKKALERLRNRFPSAAKDEVDELTNMEATVVKCCTDDDTSECISCELALAEYVCSNEYLLADELGACCKQPGE
EYTSCLKRLSTK
...

**CRITICAL:** The quality of the sequences generated by ProteinSolver will ultimately depend on the quality of the reference protein structure or distance matrix that you provide. If the generated sequences do not match your expectations, double check the provided structure or distance matrix for irregularities or typos.

**CRITICAL:** It may be advisable to try setting different values for the “temperature factor” and, based on the logos of the generated sequences, to select the temperature factor that produces sequences with the desired level of diversity. Conceptually, the temperature factor is analogous to the temperature term in a Boltzmann distribution and controls the probability that a system (in our case, a protein) will occupy a given state (in our case, the assignment of amino acids) given the energy of that state (in our case, the predictions of the ProteinSolver neural network). More concretely, the final layer of the ProteinSolver neural network is a softmax function which converts raw output values (*z*) into probabilities (*p*) of each amino acid being present at each position in the protein, and the temperature factor (*T*) scales the raw output values (*z*), thereby modifying the resulting probability distributions over the amino acids (i.e., *p*_*i*_*= exp(z*_*i*_*/ T) / Σ*_*j*_*exp(z*_*j*_*/ T)*). A large temperature factor will make the resulting distributions more flat, increasing the likelihood that residues with relatively low output values (*z*) will be selected, while a small temperature factor will make the resulting distributions more narrow, decreasing the probability that residues with relatively low output values (*z*) will be selected.

### Computational validation of the generated amino acid sequences using sequence-based tools

**Timing: 1 day**

In this step, we discuss sequence-based approaches for validating and filtering sequences generated using ProteinSolver. These approaches do not require structural models of the proteins and are especially useful in cases where it is difficult to generate a protein structure corresponding to the target topology.

8. Use PSIPRED (Jones, 1999) to predict the secondary structure of the generated sequences.
  a. PSIPRED can be accessed through a web server (http://bioinf.cs.ucl.ac.uk/psipred/) or by installing locally (see materials and equipment).
  b. Remove sequences where the predicted secondary structure does not match the secondary structure of the target protein.
9. Use ProtParam (Gasteiger et al., 2005) to predict physicochemical properties of the generated sequences.
  a. ProtParam can be accessed through a web server (https://web.expasy.org/protparam/) or using the Bio.SeqUtils.ProtParam module in BioPython (see materials and equipment).
  b. Viable protein sequences that do not aggregate under experimental conditions would generally have an Instability Index (II) less than 40 and a GRAVY coefficient between –0.5 and 0.5.
10. Use external protein structure prediction tools, such as I-TASSER (Yang et al., 2015) and QUARK (Xu and Zhang, 2012) to predict structures (with and without protein templates) of generated sequences.
  a. Use the I-TASSER web server (https://zhanglab.ccmb.med.umich.edu/I-TASSER/) to create homology models for several sequences with the highest ProteinSolver log-probability value.
  b. Use the QUARK web server (https://zhanglab.ccmb.med.umich.edu/QUARK/) to create ab-initio models for several sequences with the highest ProteinSolver log-probability value.
  c. We recommend predicting structures for only several of the highest-scoring designs, since structure prediction tools are computationally expensive.
  d. The distance matrix that was provided as input to ProteinSolver can, optionally, also be provided as input to I-TASSER. This can improve the quality of the generated structures, especially in the case of de novo design.
  e. Structural models that are produced should have distance matrices that match the distance matrix of the target topology that was provided as input to ProteinSolver.

### Computational validation of the generated amino acid sequences using structure-based tools

**Timing: 1 day**

In this step, we discuss structure-based approaches for validating and filtering the generated sequences. In order to use these approaches, we need the structure of a reference protein with the target topology, as this structure is used to construct homology models of all generated sequences. Those homology models are then evaluated using various measures of protein stability.

11. Create and evaluate homology models of the generated sequences using MODELLER (Webb and Sali, 2002).
  a. Generate alignment files in PIR file format mapping the generated sequences to the sequence of the reference protein.
  b. Create homology models of the generated sequences following MODELLER’s basic modeling tutorial: https://salilab.org/modeller/tutorial/basic.html.
  c. Obtain assessment scores for those homology models, including the GA341, DOPE, DOPE-HR and normalized DOPE scores.
12. Evaluate the stability of homology models using Rosetta (Alford et al., 2017).
  a. Relax the structures using Rosetta protocol FastRelax, as described on the Relax application page: https://www.rosettacommons.org/docs/latest/application_documentation/structure_prediction/relax.
  b. Obtain a Rosetta Energy Unit score for each structure using the ref2015 scoring function.
13. Use the obtained scores to prioritize generated sequences for more in-depth computational and experimental validation.
  a. Select one or more sequences that score best according to each of the metrics.
  b. Select one or more sequences that score best according to an average of all metrics. In order to give equal weight to each metric, before calculating the average, the scores should be normalized to have a mean of 0 and a standard deviation of 1.

### Computational validation of select amino acid sequences using molecular dynamics

**Timing: 5 days**

Molecular dynamics (MD) is arguably the least biased computational approach that can be used to evaluate the stability of *de novo* designed proteins. We suggest using MD to probe the stability of a handful of selected sequences before proceeding to experimental validation

14. Run molecular dynamics simulations using homology models constructed for to the best-scoring designs.
  a. Remove all water and ion atoms from the homology models.
  b. Solvate the structures by adding a 12 nm^3^ box of explicit water molecules, TIP3P, using the solvatebox program in AMBER16 (Case et al., 2005).
  c. Add Na+ and Cl- counter-ions to neutralize the overall system net charge using the solvatebox command in AMBER16.
  d. Generate coordinate and topology files using the tleap command (Case et al., 2005). We recommend specifying the AMBER ff14SB force field (Maier et al., 2015) when running tleap.
  e. Apply periodic boundary conditions using the sander command.
  f. Minimize, equilibrate, and heat the structures over 800 ps to 300 K, while progressively removing the positional restraints.
  g. Run the molecular dynamics simulations. We recommend using a 2 fs timestep, constraining hydrogen bonds using SHAKE (Ryckaert, Ciccotti and Berendsen, 1977), and treating long-range interactions using the particle mesh Ewald algorithm (Toukmaji et al., 2000). The simulations should be carried out for at least 30 ns and preferably more than 100 ns.
  h. Structures of the designed proteins should maintain their general topology throughout the simulations. Proteins that begin to unfold or show large per-residue RMSD values are not likely to be stable under experimental conditions.

***Note:*** While we use Amber16 in this protocol, other molecular dynamics packages can be used in its stead, including Gromacs and OpenMM.

### Experimental validation of select amino acid using circular dichroism

**Timing: 5 to 9 days**

The ultimate test for the stability and activity of the generated sequences is experimental validation. Here, we describe how to obtain circular dichroism (CD) spectra for a handful of selected designs.

15. Generate a DNA sequence and clone it into an His-tagged expression vector for E. coli purification.
  a. Reverse translate the selected amino acid sequences into DNA sequences. Make sure that the codons are optimized for E. coli and that a stop codon is added.
  b. Add restriction sites at the 5’ and 3’ ends of the reverse translated sequence for downstream cloning.
  c. Obtain the sequences commercially synthetized as a dsDNA fragment. At least 100 ng of dsDNA can be expected for each sequence. Resuspend the dsDNA to a final concertation of 10–20 μg/μl.
  d. Insert the dsDNA fragments into a His-tag expression vector by standard ligation.
16. Express the proteins. Transform the vectors containing the selected amino acid sequences into a chemically competent E. coli cells for protein expression.
  a. Plate the transformed cells into LB plates containing the appropriate antibiotic for selection.
  b. For each protein, inoculate a small spread of colonies into 15 mL of 2xYT media containing the appropriate antibiotic at 37°C, 220 rpm until the optical density (O.D.) is 0.6–0.8.
  c. Induce the cell cultures with IPTG (0.5 mM) for 3 h at 37°C.
17. Extract the soluble protein with BugBuster Master Mix (Millipore) and purify the soluble protein with Ni-NTA agarose beads (Perez-Riba and Itzhaki, 2017).
  a. Pellet the cells by centrifugation at 3000 × *g* (4°C, 10 min). Resuspend the pellets in 1 mL of BugBuster Master Mix (Millipore).
  b. Incubate the lysate mixtures for 20 min in a rotating shaker.
  c. Separate the soluble fraction by centrifugation at 16000 × *g* at 4°C for 1 min.
  d. Prepare 200 μL of Ni-NTA Agarose beads by pre-washing it in Phosphate-buffered saline (PBS) to remove the storage Ethanol.
  e. Mix the lysate supernatant with the washed beads into a 1.5 mL tube. Incubate the mixture for 20 min at 4 C in a tube rotator.
  f. Spin down the beads at 600 × *g* to remove the unbound fraction. Wash the beads with 1 mL of PBS 30 mM Imidazole.
  g. Repeat the washing step twice.
  h. After removal of the last washing step, elute the samples by addition of 1 mL of PBS, 300 mM Imidazole. Spin down at 600 × *g* to pellet the beads and keep the supernatant with the eluted protein.
  i. Dialyze the eluted sample against 5 L of fresh PBS buffer for 16 h at 4°C.

***Note:*** 1 mL of BugBuster Master Mix in our hands homogenizes a 15 mL culture of untransformed E. coli cells into a transparent solution. After centrifugation, some proteins may be present in both the soluble and insoluble fractions, and any white precipitate should be the recombinant protein in inclusion bodies. These inclusion bodies can be resuspended in Gdm HCl 6 M and further purified by Ni-NTA affinity (Perez-Riba and Itzhaki, 2017).

**Pause point:** Cell pellets obtained by centrifugation (step 17a) can be stored at –20°C for several weeks. Alternatively, purified protein may be stored at –80°C. Different proteins may require different storage conditions to preserve fold and activity.

18. Validate the purified protein sample.
  a. Calculate the protein concentration using the Pierce BCA Protein Assay Kit (ThermoFisher), Bradford assay or Tryptophan absorbance at 280 nm.
  b. Estimate sample purity by SDS-PAGE.
  c. Validate that the eluted protein matches the theoretical MW of the selected amino acid sequence by Mass Spectrometry (ESI).

***Note:*** If the protein has at least one Tryptophan, we recommend calculating protein concentration using absorbance at 280 nm. Otherwise, colorimetric assays (BCA and Bradford) are good alternatives for proteins lacking Trp residues, but the colorimetric signal is sometimes dependent on the protein chemistry. We have noticed that the same protein sample may give different concentration estimates depending on the assay used. Finally, absorbance at 205 nm can be an alternative for proteins with very low solubility (Anthis and Clore, 2013).

**CRITICAL:** Small scale purifications are cleaner in our hands than large scale ones. However, if other bands besides the recombinant protein are present in the polyacrylamide gel, other purification steps, such as gel filtrations, are required.

19. Obtain the circular dichroism spectra to estimate the secondary structure content on a CD spectrometer.
  a. Aim to have 10–20 μM of purified protein to perform CD measurements and obtain the measurements in triplicates. Using 300 μl for a 1 mm quartz cuvette is recommended.
  b. The CD spectra should be transformed to Molar ellipticity for a direct comparison between the spectra of selected amino acid sequences.
  c. Input the spectra into the BeStSel webserver (Micsonai et al., 2015) in mean residue ellipticity units to obtain predicted composition of secondary structure elements.

***Note:*** If protein dilution is required, prepare three dilution replicates and measure the protein concentration of each dilution in triplicate.

***Note:*** The Molar ellipticity is sensitive to errors in protein concentration estimation. Calculate the concentration of all the CD samples in triplicate.

***Note:*** If the concentration is below 5 uM, using a 10 mm path cuvette can improve signal, but a low salt buffer may be required to reduce the noise level.

## Expected outcomes

**Figure 2:**
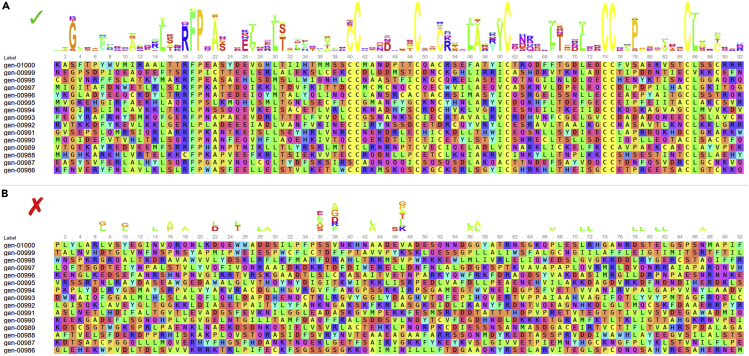
Logos comprising sequences generated to match well-defined and poorly defined topologies (A) Sequence logos comprising sequences generated to match a well-defined topology corresponding to the serum albumin protein (PDB ID: 1N5U). (B) Sequence logos comprising sequences generated to match a poorly defined topology without pairwise residue interactions.

When provided with a well-defined topology, sequences generated by ProteinSolver should show a clear preference for certain amino acids at certain positions (Figure 2A). Positions that were constrained to specific amino acids (see step 3) should be assigned only those amino acids, and the remaining positions should be filled such that the constrained amino acids are accommodated. Computational validation of the generated sequences should show that they have secondary structures that are consistent with the expected secondary structures (see step 8), physicochemical properties that are within the range observed for native sequences (see step 9), and structures that are consistent with the desired protein topology (see step 10). Computational validation of homology models obtained for the generated sequences should show that they have comparable stability to the reference structure based on a variety of metrics (see steps 11–13) and stay folded in molecular dynamics simulations (see step 14). Finally, experimental validation of the selected designs should show that the new proteins have circular dichroism spectra that are consistent with their expected secondary structures and are similar to the circular dichroism spectra obtained for a reference protein, if available (see steps 15–19).

## Quantification and statistical analysis

1.We recommend using MDTraj (McGibbon et al., 2015) for protein structure analysis, including the calculation of protein solvent-accessible surface area and secondary structure.
2.We recommend using Biotite (Kunzmann and Hamacher, 2018) for depicting pairwise alignments and secondary structure components.
3.The bootstrap method (Efron and Tibshirani, 1993) can be used to evaluate the statistical significance of measurements without assumptions regarding the underlying distribution of the data.

## Limitations

The ProteinSolver model has been validated extensively only when generating sequences for existing protein folds. In the case of de novo design, it may be difficult to come up with a distance matrix that accurately describes the topology of the target protein.

## Troubleshooting

### Problem 1

Generated sequences are too diverse. The sequence logo for the generated sequences lacks consensus (Figure 2B).

### Potential solution

Using the default temperature factor of 1 (Figure 1G), ProteinSolver will generate sequences of moderate diversity for most target topologies. In cases when only those sequences that are assigned the highest probability by the network are required, or when the target protein topology is loosely defined, it may be beneficial to decrease the temperature factor, typically 10- to 100-fold. With a lower temperature factor, ProteinSolver will sample less frequently amino acids that are assigned a lower probability by the network.

### Problem 2

Generated sequences lack diversity.

### Potential solution

In cases where the target protein geometry is exceptionally well-constrained, the sequences generated by ProteinSolver may lack diversity. One approach to increase diversity of the generated sequences is to use a higher temperature factor (Figure 1G). With a higher temperature factor, ProteinSolver will sample more frequently amino acids that are assigned a lower probability by the network. Another approach to increase diversity of the generated sequences is to first generate more sequences than are ultimately required and then cluster those sequences using some metric of sequence diversity. For example, you can use a tool such as CD-HIT or MMSeqs2 to assign sequences into clusters based on sequence identity and keep only a single sequence from each cluster.

### Problem 3

Sequences are being generated too slowly. The GPU monitoring panel shows the following error.

GPU monitoring not available ((<class 'pynvml.nvml.NVMLError_LibraryNotFound'>-

NVML Shared Library Not Found).

### Potential solution

ProteinSolver can leverage a GPU in order to increase the speed with which it generates sequences. If you are running the ProteinSolver web server locally, make sure to start the Docker container with the `--gpus all` arguments, as this is required to expose available GPUs to processes running inside the container (see materials and equipment). The GPU utilization indicator on the ProteinSolver web interface (Figure 1J) should show moderate GPU utilization when ProteinSolver is generating sequences, and GPU utilization should decrease when ProteinSolver stops. If GPU utilization is consistently at zero, it is likely that either the system is not correctly configured to use a GPU, or the version of the available GPU is not supported.

## Resource availability

### Lead contact

Further information and requests for resources and reagents should be directed to and will be fulfilled by the lead contact, Dr. Philip M. Kim (pi@kimlab.org).

### Materials availability

This study did not generate new unique reagents.

### Data and code availability

The code generated during this study is hosted on GitLab: https://gitlab.com/ostrokach/proteinsolver/. The datasets generated during this study are available at the following URL: http://deep-protein-gen.data.proteinsolver.org/.
